# Evolving use of social media among Chinese urologists: Opportunity or challenge?

**DOI:** 10.1371/journal.pone.0181895

**Published:** 2017-07-28

**Authors:** Xingbo Long, Lin Qi, Zhenyu Ou, Xiongbing Zu, Zhenzhen Cao, Xiting Zeng, Yuan Li, Minfeng Chen, Zhao Wang, Long Wang

**Affiliations:** 1 Department of Urology, Xiangya Hospital, Central South University, Changsha, Hunan, China; 2 Department of Gynecologic Oncology, Hunan Provincial Tumor Hospital and Affiliated Tumor Hospital of Xiangya Medical School, Central South University, Changsha, Hunan, China; 3 Department of Ophthalmology, Xiangya Hospital, Central South University, Changsha, Hunan, China; Carolina Urologic Research Center, UNITED STATES

## Abstract

**Background:**

Social media has revolutionized the way people communicate, and it has been widely incorporated into medical practice. However, limited data are available regarding the use of social media by Chinese urologists in their practice.

**Methods:**

From 2014 to 2016, during the China Urological Association’s (CUA) Annual National Minimally Invasive Urology Academic Conference, an anonymous survey on social media usage was distributed to participant urologists.

**Results:**

The results of the survey, which was completed by 665 participants, indicate a conspicuous increase in social media use during the last three years. Regression analysis showed that year (2014 compared to 2016 and 2015), institute location (in the eastern region of China) and age (<35 y) were independent predictors of social media use. Rather than for personal use, an increasing number of respondents said they used social media for professional purposes, and for most respondents, social media has had a positive impact on their practice. However, when posting information on social media, few respondents were aware of the issue of protecting patients’ privacy.

**Conclusions:**

Our study demonstrates a dramatic increase in social media use among Chinese urologists, which provides great opportunities for online academic communication and medical education. However, unprofessional use of social media in the medical practice may bring about potential risks and challenges for the further development of social media in medical practice.

## Introduction

With the rise of the Internet and smart phones, social media has revolutionized people’s daily lives and business interactions as well as how they develop personal and professional relationships. This new technology provides easy access to rapid networking, communication, and instantaneous consultations and lectures [1]. The limits of time and space have been overcome, and everyone can obtain desired information anytime or anywhere simply by touching their smart phones.

The use of social media by medical staff has grown substantially over the past decade. Social media, including blogs, Facebook, Twitter, LinkedIn and many other networking options, has been widely used in areas throughout medical practice, such as medical meetings and conferences[2,3], peer communication[4], medical education[5,6], patient management and education[7], public health[8], patient recruitment for studies and trials[9] and medical advertising[7]. Although emerging social media brings great merits and conveniences to medical practice, professionals and medical students alike continue to find themselves in ethical, professional, and/or legal trouble[10, 11,12].

Today, social media use is on the rise in urology, with participation from all of the major urology organizations, journals and meetings [13–16]. It has been reported that 74% of American Urological Association members [3] and 70% of Australian and New Zealand urologists[15] own social media accounts. A previous study reported a dramatic increase in the number of tweets sent at the annual meetings of the American Urological Association (AUA) and Canadian Urological Association (CUA) between 2012 and 2013[15]. Moreover, social media use was considered beneficial for networking, sharing information about research and technology, career development, and advocacy by urologists during the annual meetings of the AUA and the European Association of Urology in 2014[17,18].

However, until recently, the role of social media among Chinese urologists has been somewhat limited. Most social media platforms used in Western countries are not available in China. Chinese people have their own original social media platforms such as WeChat, QQ and RenRen (a hybrid open social media platform similar to Facebook) as well as microblog (an open social media platform similar to blogs and Twitter). The purpose of this study was to evaluate social media use among Chinese urologists and the perceived impact of social media on their practice.

## Materials and methods

Our study was approved by the ethics committee of Xiangya Hospital, Central South University. From 2014 to 2016, during the CUA Annual National Academic Conference on Minimally Invasive Urology, attendees of the meeting of urological surgeons were invited to complete an anonymous 19-item questionnaire (in 2016, 3 more questions were added to the survey, for 22 questions in total; see S1 Fig). The questionnaire was piloted by the authors and a separate group of urologists in Hunan Province based on two previous surveys[19,20]. The survey took approximately 10 minutes to complete and queried respondents about their personal and professional use of social media. The survey was randomly sent to urologists present at the conference. All of those surveyed were screened to ensure that they were active in clinical practice. Demographic information in the survey questionnaire included the respondent’s age, field of expertise, years in practice, gender, level of the hospital and institute location. In regard to institute location, we provided three options based on China’s economic geography from which participants chose: the eastern region (relatively developed areas), the western region (underdeveloped areas), and central region (between east and west).

Before the survey was closed on 10 June 2016, 200, 300 and 320 anonymous questionnaires were sent out during the CUA’s Annual Meeting on Minimally Invasive Urology in the years 2014, 2015 and 2016, respectively.

### Statistical analysis

The completed questionnaires were carefully collected and analyzed, and variables regarding respondents’ demographic information of respondents and social media use in the different years were compared using a chi-square test or Fisher’s exact test as appropriate. To determine predictive factors for having social media accounts, logistic regression analysis was used in univariate and multivariate models. Statistical tests were two-sided, and a P value <0.05 was taken as a measure of statistical significance. Data were analyzed using SPSS version 19.0. (IBM Corp., Armonk, NY, USA).

## Results

A total of 157, 242 and 266 survey questionnaires were completed, resulting in response rates of 78.5%, 80.7% and 83.1% during the years 2014, 2015 and 2016, respectively. No significant differences could be found between the years. Table 1 shows the demographics of the study population grouped by year.

**Table 1:** Characteristics of respondents stratified by year.

	Total (n = 665)	2014 (n = 157)	2015 (n = 242)	2016 (266)	Pv
Gender, n (%)†					0.77
Male	616(92.6)	147(93.6)	222(91.7)	247(92.9)	
Female	49(7.4)	10(6.4)	20(8.3)	19(7.1)	
Age, n (%)†					0.23
18–25	41(6.2)	7(4.5)	15(6.2)	19(7.1)	
26–35	130(19.5)	29(18.5)	37(15.3)	64(24.1)	
35–45	308(46.3)	74(47.1)	119(49.2)	115(43.2)	
>46	186(28.0)	47(29.9)	71(29.3)	68(25.6)	
Area of expertise, n (%)†					0.92
Urolithiasis	218(32.8)	53(33.8)	81(33.4)	84(31.6)	
Oncology	170(25.6)	40(25.5)	57(23.6)	73(27.4)	
Urinary continence	140(21.1)	34(21.7)	55(22.7)	51(19.2)	
Andrology	57(8.5)	10(6.3)	21(8.7)	26(9.8)	
Other	80(12.0)	20(12.7)	28(11.6)	32(12.0)	
Institute location, n (%)†					0.41
Eastern China	247(37.2)	66(42.0)	90(37.2)	91(34.2)	
Central China	261(39.2)	53(33.8)	100(41.3)	108(40.6)	
Western China	157(23.6)	38(24.2)	52(21.5)	67(25.2)	
Years in practice, n (%)†					0.56
Less than 5 years	86(12.9)	14(8.9)	34(14.0)	38(14.3)	
5–10 years	220(33.1)	57(36.3)	77(31.8)	86(32.3)	
10–15 years	184(27.7)	40(25.5)	66(27.3)	78(29.3)	
More than 15 years	175(26.3)	46(29.3)	65(26.9)	64(24.1)	
Level of hospital (number of beds), n (%) †					0.10
<500	110(16.5)	30(19.1)	34(14.0)	46(17.3)	
500–1000	198(29.8)	43(27.4)	87(36.0)	68(25.6)	
>1000	357(53.7)	84(53.5)	121(50.0)	152(57.1)	

^†^: Chi-square test, partitions of chi-square method is used for multiple comparisons between groups.

### Social media use increased dramatically in the last three years, and more respondents used social media for professional purposes

**Fig 1:**
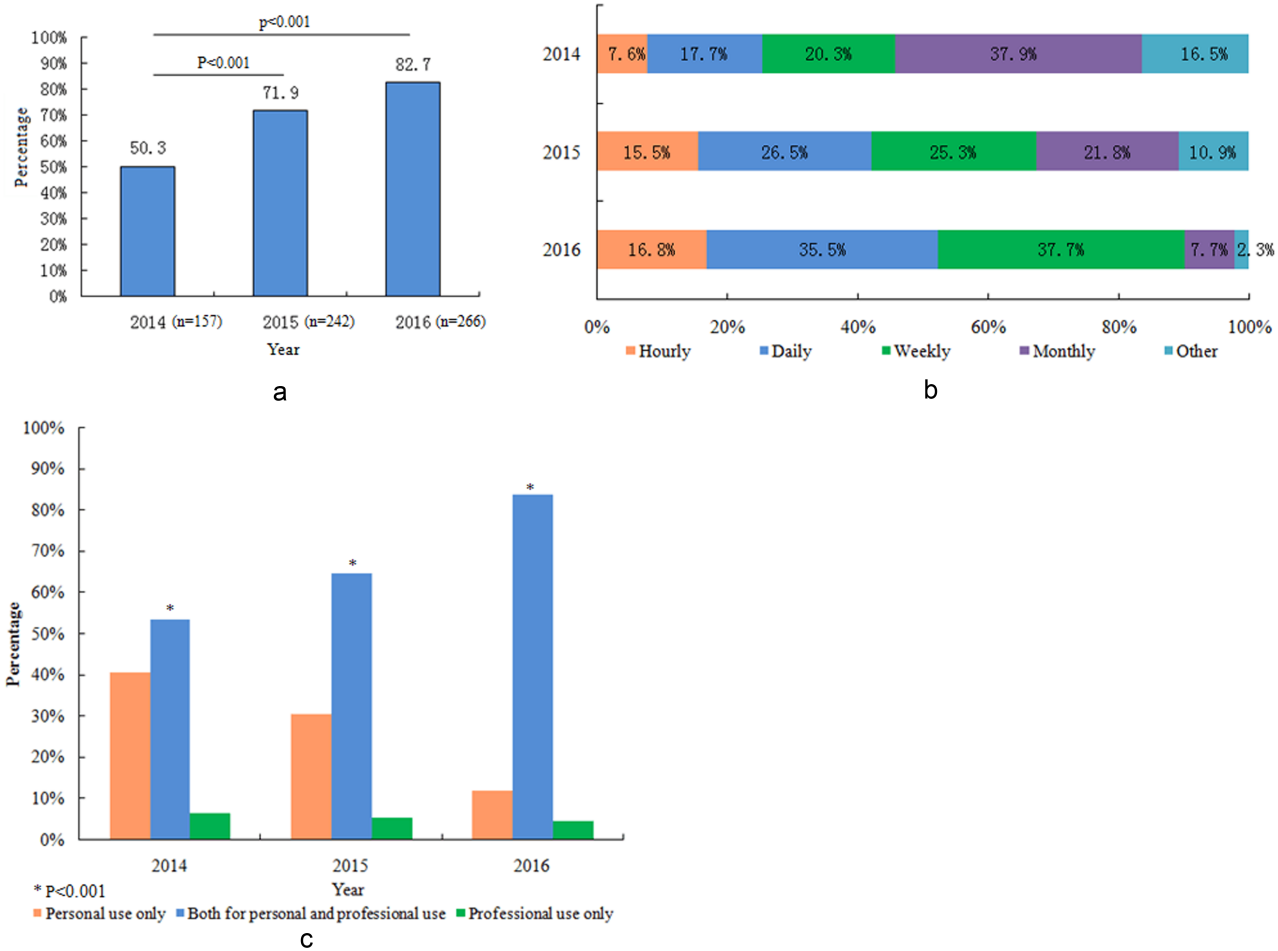
(a) The percentage of respondents using social media increased each year (p<0.001). (b) The frequency with which respondents posted messages on their social media accounts also increased each year (p = 0.001). (c) How social media accounts were used, whether professionally or personally.

There was a significant increase in the percentage of survey respondents who indicated that they have an online social media presence, which rose from 50.3% in 2014 to 82.7% in 2016 (Fig 1A, P<0.001). The survey results also suggested that the frequency with which respondents accessed their social media accounts increased over the years (Fig 1B). A regression analysis showed that year (2016 and 2015), institute location (in the eastern region of China) and age (<35 y) were independent predictors of respondents having social media accounts (Table 2).

**Table 2:** Regression analysis of predictors of having social medial account.

Predictors	Univariate			Multivariate		
	HR^‡^	95%CI^§^	P	HR	95%CI	P
Year						
2016	4.72	3.02~7.38	<0.001*	4.94	3.10~7.89	<0.001*
2015	2.53	1.66~3.85	<0.001*	2.68	1.72~4.19	<0.001*
2014(ref)	-	-	-	-	-	-
Field of expertise						
Oncology	1.19	0.65~2.18	0.57	1.35	0.7~2.60	0.38
Urolithiasis	0.74	0.42~1.30	0.29	0.69	0.37~1.29	0.25
Urinary continence	1.02	0.55~1.89	0.95	1.07	0.55~2.10	0.84
Andrology	0.77	0.42~1.89	0.89	0.74	0.32~1.71	0.48
Other(ref)	-	-	-	-	-	-
Years in practice						
less than 5 years	2.68	1.40~5.15	0.003*	0.94	0.33~2.69	0.91
5~10 years	1.46	0.95~2.25	0.088	1.02	0.47~2.20	0.97
10~15 years	1.08	0.7~1.67	0.736	0.69	0.34~1.41	0.31
More than 15 years(ref)	-	-	-	-	-	-
Institute location						
Eastern region of China	1.78	1.15~2.75	0.01*	1.91	1.16~3.13	0.011*
Central region of China	1.26	0.83~1.91	0.28	1.17	0.74~1.90	0.48
Western region of China(ref)	-	-	-	-	-	-
Gender						
Male	0.75	0.4~1.38	0.35	0.30	0.36~1.37	0.70
Female(ref)	-	-	-	-	-	-
Age						
18~25	6.97	2.07~23.44	0.002*	7.32	1.58~33.97	0.011*
26~35	2.31	1.36~3.92	0.002*	2.63	1.09~6.37	0.032*
35~45	1.18	0.80~1.73	0.40	1.34	0.67~2.68	0.41
>46(ref)	-	-	-	-	-	-
level of hospital						
500–1000	1.39	0.84~2.30	0.21	1.48	0.86~2.55	0.16
>1000	1.27	0.80~2.00	0.31	1.43	0.86~2.38	0.17
<500(ref)	-	-	-	-	-	-

^‡^ HR: hazard ratio

^§^95%CI: 95% confidence interval

*Statistically significant at P<0.05.

Of all the respondents with an online social media account, WeChat accounts were the most common (92.8%), followed by QQ (83.5%), Weibo (63.0%), RenRen (26.0%), and LinkedIn (11.2%). No significant differences could be found between the years.

In regard to the question about what their social media accounts were used for, Fig 1C shows that, since 2014, an increasing number of respondents have become willing to use social media both professionally and personally, instead of only for personal or professional use (P<0.001).

### Professional use of social media

**Fig 2:**
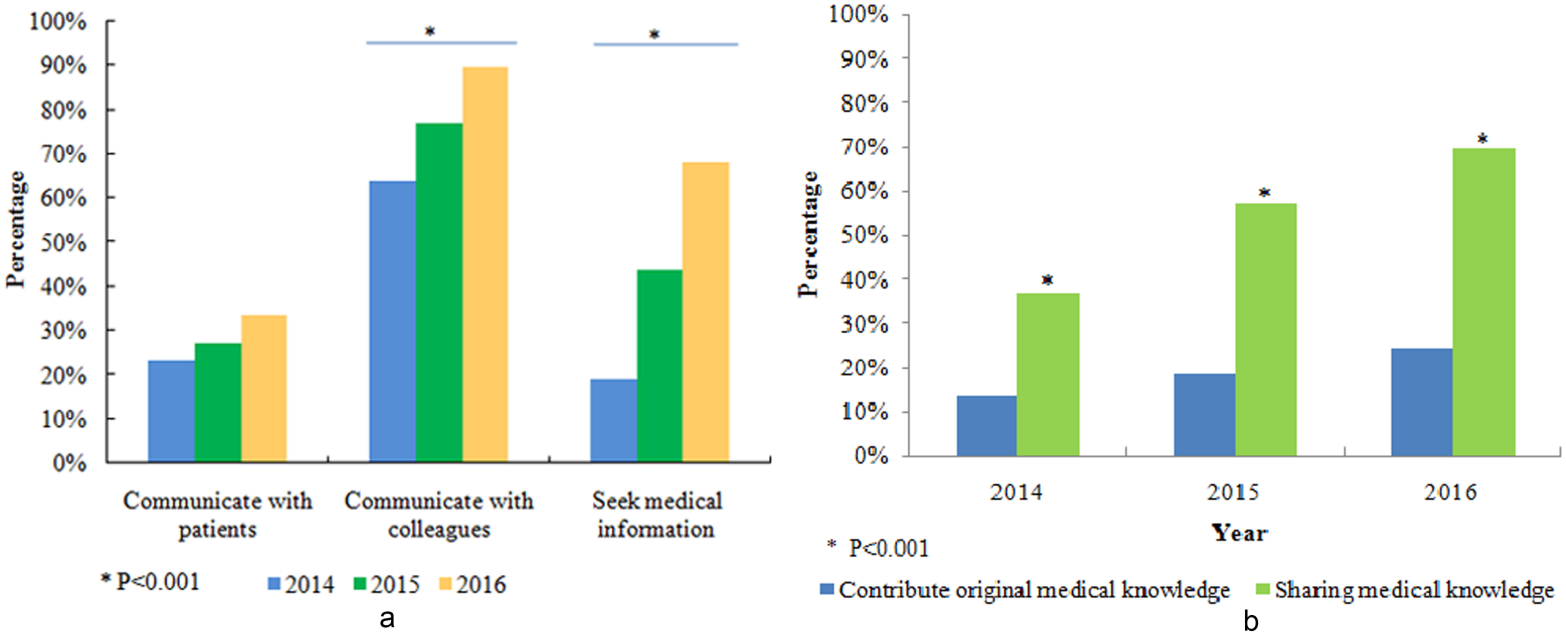
(a) Regarding the professional use of social media, the percentage of respondents using social media in medical education or communication with colleagues or seek medical information increased dramatically each year (p<0.001), while the percentage of respondents using social media to communicate did not change much over the three years. (b) In academic communication over social media, respondents were more willing to share and pass on information than to provide original information.

Among the respondents who used social media accounts professionally, only approximately 30% used social media accounts to communicate with patients, and this percentage did not increase much during the last three years (Fig 2a). Meanwhile, a dramatic increase was found in the percentage of respondents using social media accounts for surgical or medical education or for communication with peers and colleagues. Furthermore, respondents were more and more willing to use social media to seek specific information when facing a medical problem or situation. In 2014, less than 20% of respondents had experienced seeking specific information through social media, and that percentage rose dramatically to more than 60% in 2016 (Fig 2a). Although academic communication became increasingly popular on social media, respondents seemed more willing to share and pass on medical knowledge than to contribute original information (Fig 2b).

The reasons for which respondents used social media accounts professionally were various. The most common reason was to improve networking or collaboration with peers and colleagues (66.7%), followed by the belief that social media provides a platform for surgical or medical education (52.7%). Facilitating more efficient communication with patients was the third most common reason (32.7%), and just 17.5% of respondents regarded social media as an effective marketing or advertising tool when used professionally. After analyzing the trends in social media use over the past three years, we have observed that the value of social media as a medical education platform has been realized by more and more respondents, except for this, no significant differences could be found between the years.

In terms of communication with patients, the majority of respondents thought social media had improved efficiency in patient education (65.4%) and patient communication (55.1%). Only 34% and 29% of respondents received unwanted solicitation and thought it took too much time, respectively. No significant differences could be observed between the years. What should not be ignored is that, although only 17.6% respondents thought social media provided a low-cost means of advertising in 2014, this percentage increased to 51.5% in 2016 (P<0.001).

### The impact of social media on medical practice

**Fig 3:**
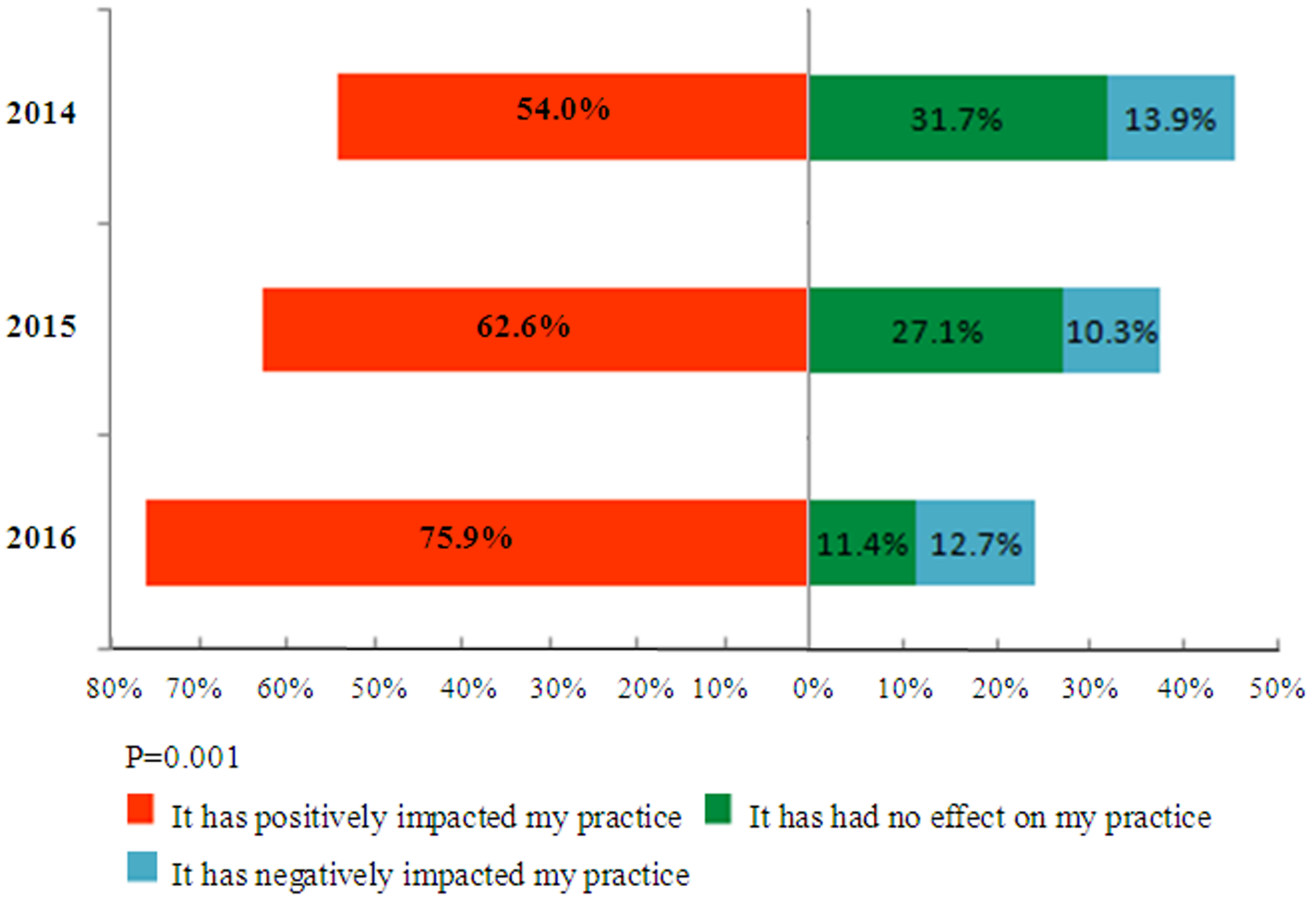
For most respondents, during the last three years, social media has had a positive impact on their practice.

As Fig 3 shows, in the last three years, an increasing number of respondents reported that social media has had a positive impact on their practice (p = 0.001), and only approximately 10% reported a negative impact on their practice. For those who observed a positive impact on their practice, most answered that social media improved their effectiveness in surgical or medical education (68.3%), increased the exposure of their practice locally, regionally, and beyond (62.7%), and was a source of positive feedback from patients by means of these platforms (32.4%). Only a small number of those queried felt that social media has allowed them to be more efficient in their communication with patients (20.5%) or has provided them with a low-cost means of advertising (25.8%).

Those who observed social media as having a negative impact on their practice complained that time spent on social media took time away from clinical practice (75.8%) and resulted in unwanted solicitations from advertisers (58.7%). Moreover, 38.4% indicated that negative comments by patients have hurt their practice and referral base. Only a few respondents claimed social media is too costly (12.5%) and felt that patient privacy had been compromised (8%).

### Caution advised about unprofessional social media use in the medical practice

Nearly half of the respondents had experience posting information or pictures of patients on social media, but only 5% of them sought their patients’ consent before posting. More surprisingly, although academic communication has become increasingly popular on social media, few respondents(6%) reported that they confirmed the reliability and accuracy of the information before providing and sharing it on social media platforms.

## Discussion

Whether we accept it, the era of social media is here to stay. It is the snowball at the top of an emerging avalanche whose development and widespread use is inevitable. The question for surgeons is not whether to use it, but how to use it. For the naysayers, it is time to take a new view of social media in the context of what it can and cannot provide for surgeons and patients alike.

During the last three years, from 2014 to 2016, our study has witnessed a conspicuous increase in social media use among Chinese urologists. For most respondents, social media has had a positive impact on their practice. In accordance with previous studies[3,21,22], our study found that the social media accounts of young respondents (under 35 years of age) have higher levels of social media engagement than those of older respondents. We also found that economic-geographic location was an independent predictor of whether respondents had social media accounts; respondents from relatively developed areas (the eastern region of China) were more likely to have social media accounts than those from underdeveloped areas (the western region of China).

Unlike a previous study implemented among AUA members in which only 28% of respondents indicated that they used social media accounts for professional purposes[3], in our study, even in 2014, over 50% respondents declared that they used social media professionally, and that number increased to over 80% in 2016. Among the respondents who declared that they used social media professionally, there was a dramatic increase in the percentage of respondents who used their social media accounts for surgical or medical education or communication with peers and colleagues. Meanwhile, improved networking or collaboration with peers and colleagues was one of the main reasons why respondents chose to use their social media professionally.

With the development and widespread adoption of mobile smartphones and computers, social media possesses distinctive advantages in the rapid and efficient exchange and delivery of information, and its power as a means of exchanging medical information has been reported by a previous prospective mixed-methods study among emergency surgical teams[4]. That study demonstrated that, compared with the old-fashioned method of phone calls, social media helped to remove communication barriers between junior and senior colleagues and gave them the ability to send a quick message. For senior colleagues, social media increased the level of supervision on their team and allowed them to react quickly to patients’ pathogenetic conditions.

Apart from the exchange of clinical information, social media has also been widely used in academic communication, especially in meetings or conferences [2.3], as it allows people to overcome regional limits and enables people from all corners of the country to join or even participate in a meeting. In China, WeChat live broadcast technology has been widely applied in national academic conferences. During our 2016 CUA Annual National Academic Conference on Minimally Invasive Urology, Over 100,000 people watched the online live broadcast of the conference through social media platforms—an audience 200 times larger than the number of people attending the conference.

In addition to academic or peer communication, it is well known that social media platforms, such as Facebook and Twitter, have been introduced into medical education at colleges and universities in Western countries[23], and the role of social media in medical education has been recognized by an increasing number of medical personnel. In our study, more than 50% of respondents thought social media provided a platform for surgical or medical education, and respondents were more and more willing to use social media to seek out specific information when facing a medical problem or situation. Although academic communication has become increasingly popular on social media, respondents seem more willing to share and pass on medical knowledge than contribute original information.

Compared to communication with peers and colleagues, in our study, merely approximately 30% of respondents were willing to use their social media to communicate with patients. Meanwhile, a smaller number of respondents who used social media professionally felt that social media has allowed them to be more efficient in their communication with patients (20.5%) or provided them with a low-cost means of advertising (25.8%). This situation is quite different from the findings of previous studies in Western countries[24,25], in which social media played an important role in patient management and recruitment as well as advertising, especially for plastic surgeons. Some social media platforms allow patients going through similar experiences to communicate with each other to provide support and camaraderie, which in return would enhance the compliance of those patients[26]. A recent prospective multicenter study in China of 770 colonoscopy outpatients found that use of a social media app to educate patients before colonoscopy significantly increased the proportion of patients with a high quality colonoscopy[27]. Additionally, in our study, the majority of respondents who used social media to communicate with patients thought that social media had improved their efficiency in patient education and communication. However, patient management via social media requires patients with higher levels of education, and in China, most patients may not meet the requirements. This may be one of the reasons why only a minority of respondents had experience in communicating with patients over social media.

Although social media has many merits and can bring many conveniences to our daily medical practices, there are many potential pitfalls of which medical professionals should be aware. Unlike the situation in North America and Europe, where several social media policies and guidelines exist[28–30], so far, in China, there are no clear, concise, and currently relevant media policies available to guide our social media activities in the medical practice. Although the clause that protects health information and patient privacy should never be compromised by any form of communication according to China’s latest Management Measures for Medical Quality (2014), most Chinese medical staff are not familiar with this policy and continue to post information or pictures of patients on social media unintentionally and without any punishment. In our study, only 5% of respondents sought patients' consent before posting information or pictures of patients on social media. Respondents also seldom reported that they confirmed the reliability and accuracy of information before providing and sharing it on social media platforms. Unprofessional social media use in the medical practice may mean problems for the further development of social media in medical practice.

Several limitations of the present study must be mentioned. Although the survey was distributed to a random sample of more than 800 attendees, the response rate was approximately 80%. There may be systematic differences between those who did and did not reply to the survey. Another limitation of the present study is that our research subject is limited to the attendees of the CUA Annual Meeting on Minimally Invasive Urology, and urologists who did not participate in the meeting were not included in the study, which could inevitably bring some selection bias.

## Conclusions

Our study demonstrates a dramatic increase in social media use among Chinese urologists, something that provides great opportunities for online academic communication and medical education. However, the unprofessional use of social media in the medical practice may bring about potential risks and challenges for the further development of social media in medical practice.

## Supporting information

S1 Fig The questionnaire. (PDF)
