# Supplementary material for: Evolving use of social media among Chinese urologists: Opportunity or challenge?
Source: PLoS One. 2017 Jul 28;12(7):e0181895. doi: 10.1371/journal.pone.0181895 (PMC5533312; doi:10.1371/journal.pone.0181895)
Supplement: S1 Fig — (PDF) [file pone.0181895.s001.pdf]

Questionnaire

Ⅰ The basic information

1. Please indicate your age： 18-25/ 26-35/ 35-45/ >46

2. Are you: Female /Male

3. What’s your area of expertise? Oncology/ Urolithiasis/ Urinary continence/ Andrology/ Other

4. How many years have you been in practice? less than 5 years/ 5-10 years/ 10-15 years/ More than 15 years

5. Where is your institute located in? In the eastern china (Relatively Developed Areas)/ central region of China/ western region of china (Underdeveloped Areas)

6. Do you currently have a social media account? (Y/N), if yes, which platforms do you actively use? (Check all that apply) WeChat/ weibo/ QQ/ Renren/ LinkedIn/ Other

Ⅱ If you have social media accounts, please answer the following questions

7. How often do you post massages to your social media account? Hourly /Daily/ Weekly/ Monthly/ Other

8. Do you use social media personally or professionally? only for personal use/ only for professional use/ for both personal and professional use

Ⅲ If you use social media professionally, please answer the following questions

9. If you do use social media in your professional, can you tell us why? (Check all that apply) Effective marketing or advertising tool/ Provides a platform for surgical or medical education. /Improves networking & collaboration with peers and colleagues/ Facilitates more efficient

communicate with patients/ Fellow other peers or colleagues and feel incorporation of social media into medical practice is inevitable/ Other.

10. Do you use your social media account to communicate with patients?   Y/N (If yes, please answer the question 11)

11. When using social media account to communicate with patients, how you evaluate it. It has improved my effectiveness as a patient educator/ It has improved my efficiency with patient communication/ It has allowed a low-cost means of advertising/ It has resulted in unwanted solicitations from patients/ it cost too much time to communicate with patients.

12. Have you ever used social media in surgical or medical education or peers and colleagues communication? Y/N

13. Have you ever contribute original medical knowledge to social medial? Y/N (If yes, please answer the question 15)

14. Have you ever sharing and passing medical knowledge to social medial? Y/N (If yes, please answer the question 15)

15. When providing, sharing and passing information on the social media, have you ever confirmed the information is reliable and accurately. Never/ Sometimes/ Always (The question was added to the survey in the 2016)

16. Have you ever using social media to seek specific information about a medical problem or situation? Y/N (If yes, please answer the question 17)

17. Did you feel the information you gain from social media is useful? Y/N

18. Have you ever posed information or pictures of patients on social media? Y/ N (If yes, please answer the question 19. The question was added to the survey in the 2016)

19. Before posing information or pictures of patients on social media, have you sought for patients' consent? Never/ Sometimes/ Always (The question was added to the survey in the 2016)

20. Which of the following best describes your experience with social media in the past year? It has positively impacted my practice/ It has negatively impacted my practice/ It has had no effect on my practice.

21. If social media has had a positive impact on your practice, can you indicate how? (Check all that apply) It has increased the exposure of my practice locally, regionally, and beyond/ It has improved my effectiveness as a surgical or medical education/It has improved my efficiency with patient communication/ It has allowed a low-cost means of advertising/ I have received positive patient feedback through social media websites/ Other

22. If social media has negatively impacted your practice, can you indicate how? (Check all that apply) Negative patient comments have hurt my practice and referral base/ Time spent on social media takes time away from clinical practice/ It has resulted in unwanted solicitations from advertisers/ Too costly/ Patient privacy has been compromised/ Other.
